# Research status and hotspots of the primary health care for chronic disease: A bibliometric and visualized analysis

**DOI:** 10.1097/MD.0000000000049383

**Published:** 2026-06-26

**Authors:** Ke Li, Chengquan Lu, Jian Wang

**Affiliations:** a Department of General Practice Clinic, Jinzhou Hospital of Traditional Chinese Medicine Jinzhou Third Hospital, Jinzhou, Liaoning Province, China; b Department of Immunization Program, Jinzhou Center for Disease Control and Prevention, Jinzhou, Liaoning Province, China; c Department of AIDS and STD Prevention and Control, Jinzhou Center for Disease Control and Prevention, Jinzhou, Liaoning Province, China.

**Keywords:** bibliometric study, chronic disease, comprehensive health care, multiple chronic conditions, primary care

## Abstract

**Background:**

This study aims to explore the global research trends, hotspots, and core characteristics of primary health care for chronic diseases from 2014 to 2024 via bibliometric methods, providing a comprehensive reference for scholars, policymakers, and public health practitioners.

**Methods:**

We retrieved relevant publications from the Web of Science Core Collection and Science Citation Index Expanded databases covering 2014 to 2024, and included 3435 valid English articles and reviews after screening. VOSviewer and CiteSpace were used to analyze publication output, geographical distribution, institutions, journals, authors, co-cited references, and keyword co-occurrence clustering.

**Results:**

Annual publications showed a steady growth trend. The United States was the leading contributing country, the University of Toronto ranked as the most productive institution, and BMJ Open published the most relevant papers. The most cited article by De Onis M (2016) had a global citation score of 665. Keyword clustering identified 2 core themes: chronic disease epidemiology and the effectiveness of primary care management models.

**Conclusions:**

This review clarifies the global research status, core contributors, and key themes in this field. Future research will focus on innovative management strategies, societal participation, and internet technology integration. The findings provide evidence-based insights for decision-making to reduce chronic disease incidence and improve patient outcomes and health-related quality of life.

## 1. Introduction

Since the publication of the Alma-Ata Declaration over 40 years ago in 1978, global health goals have evolved from “Health for All by the Year 2000” to Universal Health Coverage.^[1]^ Primary health care (PHC) remains the fundamental approach to achieving global health strategies.^[2]^ A well-established general practice service system is not only a hallmark of a successful PHC system but also a key indicator of a thriving national health system.^[2]^ Internationally, general practitioners (GPs), who serve as the first point of contact without requiring referrals, have a long history. Marking the founding of the Royal College of General Practitioners in the United Kingdom as a milestone, the professionalization of GPs has been ongoing for over 60 years.^[3]^ In countries with well-developed general practice (family medicine) services, such as the United Kingdom, Canada, the United States, Australia, New Zealand, and the Nordic countries, GPs are central to the national and regional healthcare systems, acting as the first point of contact without the need for referrals. These nations have made significant strides in establishing mature general practice service systems. For example, Australia initiated universal health insurance in the 1970s and formally established general practice training programs, with medical schools setting up general practice departments.^[4]^ However, in developing countries, the construction of general practice (family medicine) service systems still requires considerable strengthening.^[5]^

From 2000 to 2021, global disease burden data, measured by disability-adjusted life years, indicate that the leading causes of mortality and morbidity are ischemic heart disease, stroke, chronic obstructive pulmonary disease (COPD), diabetes, and lower back pain (https://vizhub.healthdata.org/gbd-compare/). In high-income countries, the primary causes are ischemic heart disease, lower back pain, diabetes, Alzheimer disease, and lung cancer (https://vizhub.healthdata.org/gbd-compare/). It is evident that the burden of chronic diseases is significant worldwide, with many individuals suffering from multiple chronic conditions, referred to as multimorbidity.^[6]^ The term “comorbidity” is also used, but it typically refers to a specific index condition associated with other conditions, such as diabetes and cardiovascular disease, or combinations of commonly co-occurring diseases, like diabetes and depression.

Estimates of the prevalence of multiple chronic conditions are highly heterogeneous due to differing methodologies, including the number of chronic diseases counted, leading to a threefold variation in estimates.^[7,8]^ The US Centers for Medicare and Medicaid Services define criteria for All-Cause Unplanned Admissions for Patients with Multiple Chronic Conditions as follows: individuals aged 66 and older with International Statistical Classification of Diseases and Related Health Problems, 10th Revision codes belonging to 2 or more of the following 9 chronic disease groups: acute myocardial infarction, Alzheimer disease and related disorders, atrial fibrillation, chronic kidney disease, COPD and asthma, depression, heart failure, stroke and transient ischemic attack, and diabetes.^[8]^ Multimorbidity is a major issue in primary care, with determinants including age, lower socioeconomic status, and gender. The most prevalent conditions form patterns of multimorbidity, with common combinations including osteoarthritis and cardiovascular and/or metabolic diseases.^[9]^

Garin N et al analyzed data from the Collaborative Research on Aging in Europe (Finland, Poland, and Spain) and the World Health Organization’s Study on Global Ageing and Adult Health (China, Ghana, India, Mexico, Russia, and South Africa), revealing a high overall prevalence of multimorbidity across countries. Hypertension, cataracts, and arthritis were the most common comorbidities, identifying patterns such as “cardio-respiratory” (angina, asthma, and COPD), “metabolic” (diabetes, obesity, and hypertension), and “mental-articular” (arthritis and depression).^[10]^ Although not a new phenomenon, the impact of multimorbidity and the importance of addressing it for better patient outcomes are increasingly recognized. Addressing this issue requires substantial health resources, including medications, primary care, outpatient specialty services, emergency department visits, and hospitalizations. The utilization of PHC varies significantly across different countries and regions.^[7]^

To date, there has been considerable research on PHC for chronic diseases, particularly in the context of multimorbidity, but the research is highly heterogeneous. A bibliometric analysis is urgently needed to quantify the current status, key areas, and future prospects in this field. Therefore, this study aims to conduct an in-depth investigation using bibliometric methods to assess the status and research hotspots in the field of PHC for chronic diseases.

## 2. Methods

This study is a cross-sectional bibliometric analysis that incorporates scientific knowledge mapping techniques, aimed at systematically reviewing the research landscape and emerging trends in the field of chronic disease PHC from 2014 to 2023. The data analysis process is divided into 3 stages: Data Retrieval and Cleaning: raw data were obtained from the Web of Science Core Collection (WoSCC; including sub-databases such as Science Citation Index Expanded (SCI-E) and Social Sciences Citation Index); Literature Screening and Classification: studies were selected according to predefined criteria, and relevant fields such as country, institution, author, journal, and keywords were extracted; Visualization and Trend Analysis: VOSviewer (1.6.19; Centre for Science and Technology Studies, Leiden University) was used to construct co-occurrence networks, while CiteSpace (6.2.R4; Chaomei Chen, Drexel University) was employed for keyword burst detection and timeline analysis. This study was strictly designed and reported in accordance with the BIBLIO_Checklist.

Ethical approval was not required for this bibliometric review. This study is a bibliometric analysis focused on publicly available academic publications retrieved from the WoSCC and SCI-E databases. All data used in this research are derived from secondary analysis of existing, peer-reviewed, and publicly accessible scientific literature, which does not involve human subjects, patients, or any identifiable personal information (e.g., personal demographic data, medical records, or private information of individuals).

As the study does not involve direct or indirect interaction with human participants, the collection of personal data, or any interventions that may affect the rights and interests of individuals, it does not meet the criteria requiring ethical review as stipulated by relevant ethical guidelines. In addition, no informed consent was needed for this study, as there are no human subjects or patients involved, and the research process does not require the participation or authorization of any individual.

### 2.1. Sources of information and search methodologies

The literature data set was compiled using the WoSCC, which provides a comprehensive and standardized set of data for export and is widely used in academia. The literature search was conducted in 1 day (May 22, 2025) to avoid deviations, taking into account the rapid update of the database. The following were the search terms: ((TS=(“primary care”) OR TS=(“Primary Health Care”) OR TS=(“Comprehensive Health Care”)) AND ((TS=(“Chronic Disease”) OR TS=(“Multiple Chronic Conditions”)). Among the various types of publications, only English-language articles and reviews were considered. In total, 3435 articles were ultimately analyzed from 2014 to 2024. The results of the thorough screening may be found in Table 1. Inclusion criteria: studies explicitly addressing the relationship between chronic diseases (such as diabetes, hypertension, and arthritis) and PHC, original research articles or reviews published in English, and studies published between 2014 and 2024. Exclusion criteria: edited materials, conference abstracts, proceedings, and other non-research literature published in non-English languages. The screening process was carried out independently by 2 reviewers, and any discrepancies in the selection were resolved by a third reviewer.

**Table 1:** TS search queries and screening process.

Set	Search query	Results
1	TS=(“primary care”)	187,334
2	TS=(“Primary Health Care”)	35,479
3	TS=(“Comprehensive Health Care”)	1058
4	#1 OR #2 OR #3	210,457
5	TS=(“Chronic Disease”)	58,450
6	TS=(“Multiple Chronic Conditions”)	2480
7	#5 OR #6	60,517
8	#7 AND #4	5475
9	#7 AND #4 and 2024 or 2023 or 2022 or 2021 or 2020 or 2019 or 2018 or 2017 or 2016 or 2015 or 2014 (Publication Years)	3676
10	#7 AND #4 and 2024 or 2023 or 2022 or 2021 or 2020 or 2019 or 2018 or 2017 or 2016 or 2015 or 2014 (Publication Years) and Article or Review Article (Document Types)	3512
11	#7 AND #4 and 2024 or 2023 or 2022 or 2021 or 2020 or 2019 or 2018 or 2017 or 2016 or 2015 or 2014 (Publication Years) and Article or Review Article (Document Types) and English (Languages)	3435

Database: Web of Science Core Collection.

Date Run: May 22, 2025.

### 2.2. Data collection

Complete records of the selected literature (including references, citation counts, author affiliations, etc) were exported from Web of Science. Duplicates were removed using the built-in functionality of VOSviewer, and studies with missing key information (such as absent abstracts or author details) were excluded. Data for all identified articles were obtained from the database “Web of Science Core Collection,” and the data were collected, including authors, affiliations, countries/regions, journals, the number of papers and citations, publication year, H-index, keywords, and references. Two writers independently browsed the qualifying papers and extracted data. The data were then analyzed further using an online program (http://www.bioinformatics.com.cn/), VOSviewer v1.6.10.0, and CiteSpace (version 5.8.R3).

### 2.3. Bibliometric analysis

The bibliometric analysis was carried out using VOSviewer (version 1.6.10), CiteSpace (version 5.8.R3), and the web tool (https://www.bibliometrix.org). The number of publications (Np) and citations (Nc) often used to signify bibliographic materials are examples of bibliometric indicators. As the 2 essential viewpoints for measuring research performance, the Np is often used to quantify productive capacity, and the Nc can demonstrate impact. The *H*-index is primarily used to assess researchers’ academic contributions and forecast future scientific accomplishments.

The *H*-index unites productivity and influence by identifying a threshold that links Np and Nc. A researcher will have an *H*-index if they have produced H articles, each quoted at least H times. Besides, the *H*-index was designed to assess personal academic performance. However, it may also now define a country’s or region’s publishing output and an institution’s or journal’s production.^[11,12]^

In addition, the impact factor (IF) calculated from the most recent edition of the Journal Citation Reports is widely recognized as one of the most important indices of medical journal quality and impact.^[13]^ The global citation score (GCS) is considered the Nc of an article worldwide. It is an essential indicator of the contribution an article makes to the field of knowledge, with a high GCS indicating a high level of interest from scientists worldwide.^[14]^ The fitted polynomial model was used to forecast the yearly Np further to demonstrate the variations in the annual publishing amount. The yearly number of investigations is represented by variable f (x), and the publication year is indicated by x.

VOSviewer v1.6.10.0 is used to construct and visualize bibliometric network graphs (Leiden University).^[15,16]^ VOSviewer was used to perform co-citation and co-occurrence analysis in this study. The size of the nodes represented the Np, the thickness of the line showed the strength of the relationship, and the colors of the nodes represented distinct clusters or periods. VOSviewer 1.6.19 Parameter Configuration: we imported raw data from Web of Science as full records and references in .csv format, and chose the option to “Create a map based on bibliographic data.” The Full Counting method was applied for association strength calculation, with the following thresholds: a minimum of 15 citations for a referenced source and 30 occurrences for a keyword. The VOS clustering technique was used with a resolution of 1.0, resulting in 5 to 8 clusters. Layout optimization parameters included an attraction value of 2, a repulsion value of 0, and 200 iterations to ensure stability. Node sizes were proportional to keyword frequency or institutional publication output, while node colors were assigned based on clusters. We also limited the number of displayed keywords to the top 50 by frequency to avoid label overlap.

Cluster analysis, timeline or time zone views, references, and keyword citation bursts were all employed in CiteSpace to aid in the visual evaluation of knowledge fields and developing trends.^[17]^ Cluster analysis enables the categorization of references and keywords, as well as the discovery of essential study topics for GA. Bursts of keywords and references are frequently utilized to discover new research trends. CiteSpace 6.2.R3 Parameter Configuration: data were exported from Web of Science in plain text format (.txt), including full records and references. The time slice was set to 1 year, spanning from 2014 to 2024, and the nodes analyzed included keywords and cited references. The threshold for keywords was set to a frequency of 20 or more, and for cited references, a citation count of 50 or more. We used the Pathfinder algorithm to prune weak connections and applied a slice-by-slice pruning method for redundant connections. We employed the log-likelihood ratio method for clustering and set a minimum burst strength of 3.0 for burst detection. The Timezone View displays the annual distribution of keywords and references, with node size reflecting frequency and color indicating the year of first appearance. The Timeline View illustrates the evolution of research themes by cluster.

## 3. Results

### 3.1. An overview of publications on PHC for chronic disease

**Figure 1.**
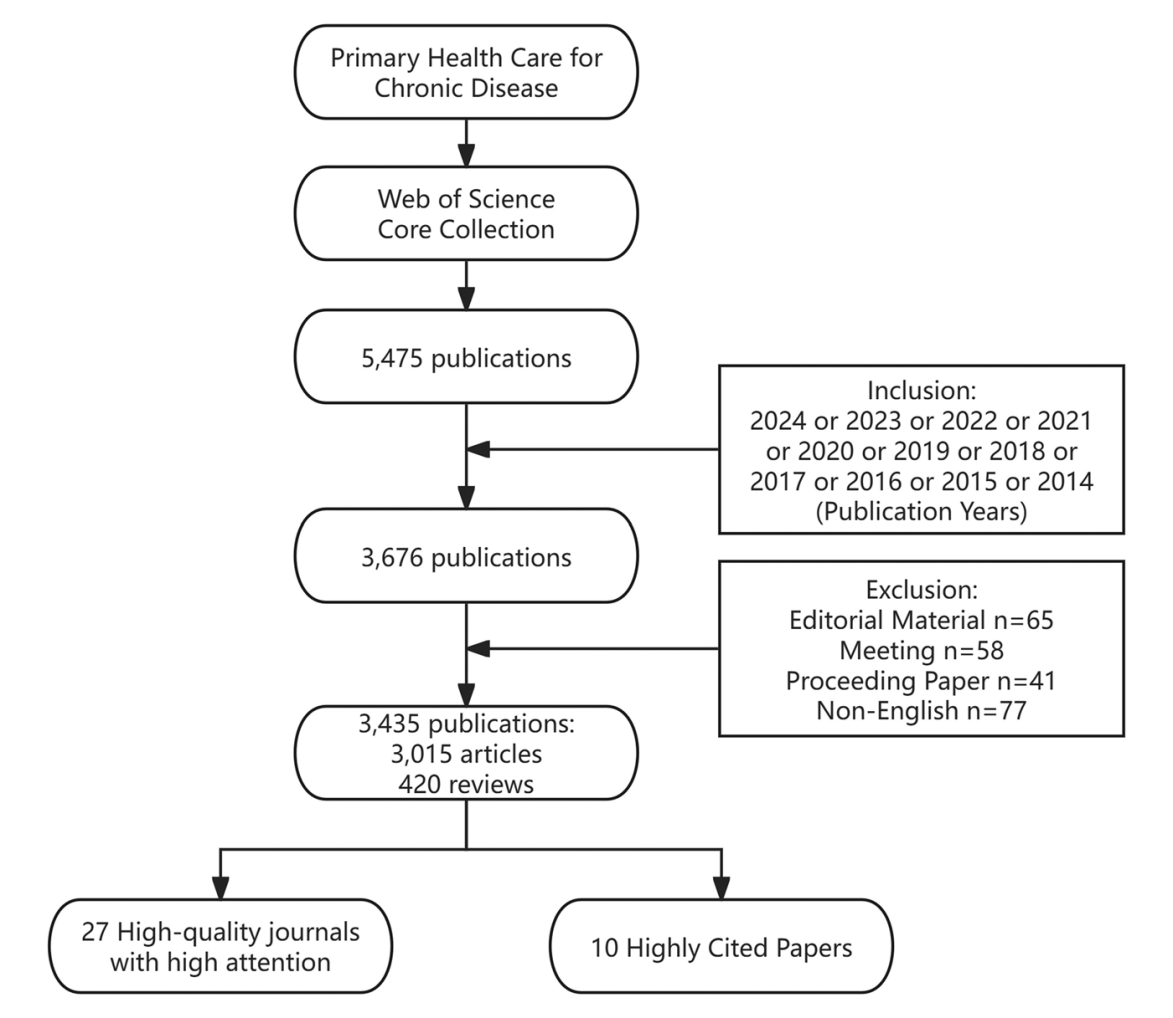
Flowchart of the research.

A search in the WoSCC yielded 3435 articles and reviews published from 2014 to 2024, including 3015 articles and 420 reviews. The search query used was: (TS=(“primary care”) OR TS=(“Primary Health Care”) OR TS=(“Comprehensive Health Care”)) AND (TS=(“Chronic Disease”) OR TS=(“Multiple Chronic Conditions”)), resulting in 5475 documents. Refining by publication year (2024, 2023, 2022, 2021, 2020, 2019, 2018, 2017, 2016, 2015, and 2014) yielded 3676 documents. Further refining by document type (Article or Review Article) narrowed it down to 3512 documents. Finally, refining by language (English) resulted in 3435 documents. Detailed data can be found in Table 1. The flowchart is shown in Figure 1. The total Nc value of the retrieved documents is 55,074, with an average Nc value of 18.46. The *H*-index of all publications is 98.

**Figure 2.**
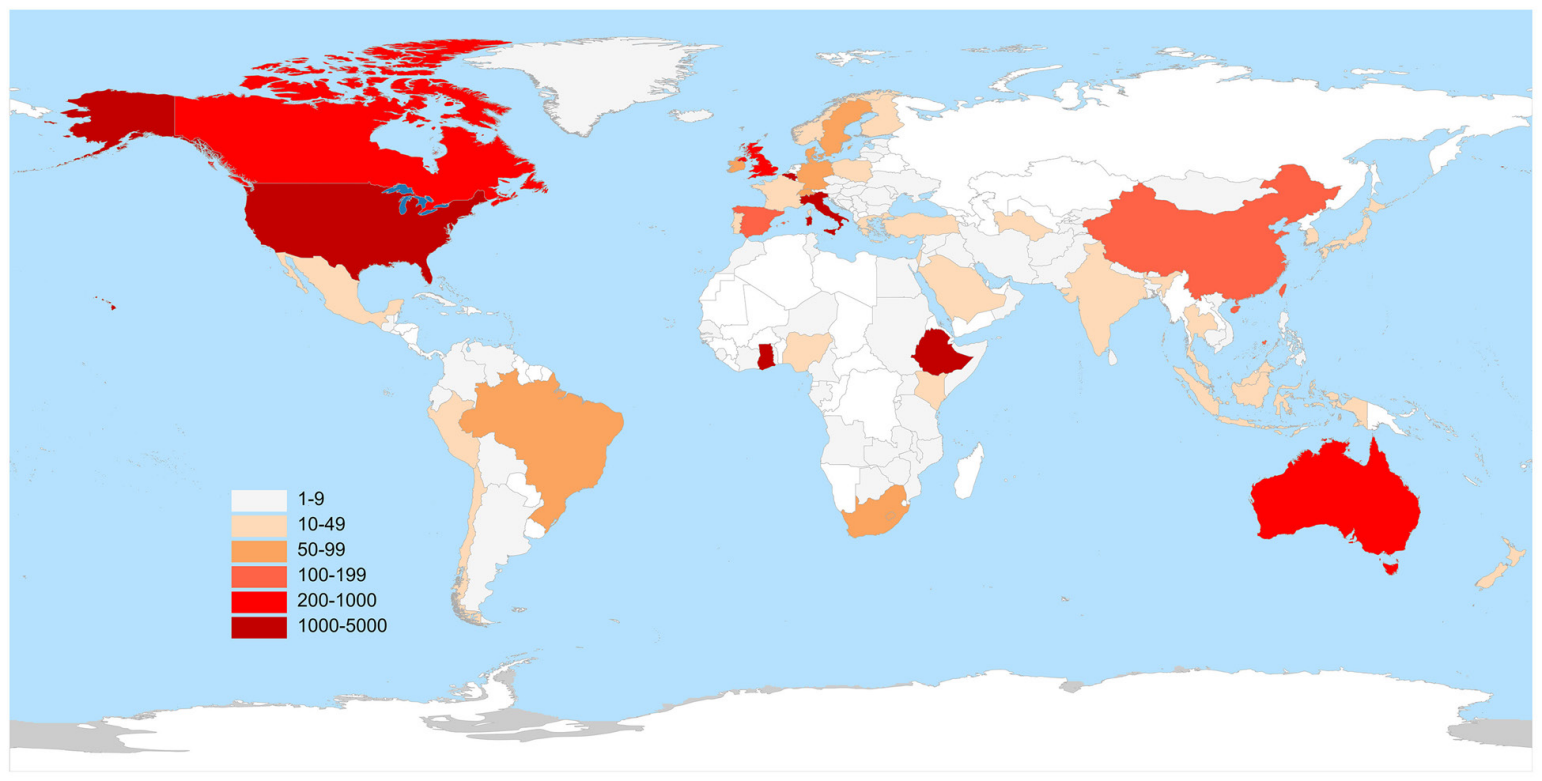
Geographical distribution of publications on primary health care for chronic disease research, 2014 to 2024.

Analysis of the geographical distribution of publications (Fig. 2, Table 2) reveals that the top 5 countries account for 82.13% of the 3435 articles. The United States leads with the highest Np, followed by Canada, Australia, England, and China. This distribution highlights the significant contribution of these countries to research in PHC for chronic diseases.

**Table 2:** The top 10 countries/regions with the highest productivity.

Rank	Countries/regions	Np	% of (3435)	Nc	*H*-index
1	USA	1344	39.13	23,752	70
2	CANADA	483	14.06	11,756	53
3	AUSTRALIA	475	13.83	9066	45
4	ENGLAND	346	10.07	10,229	56
5	CHINA	173	5.04	2115	25
6	SPAIN	156	4.54	4345	31
7	NETHERLANDS	153	4.45	6125	39
8	BRAZIL	76	2.21	896	11
9	GERMANY	73	2.13	1828	18
10	SOUTH AFRICA	73	2.13	1582	27

Nc = number of citations, Np = number of publications.

**Figure 3.**
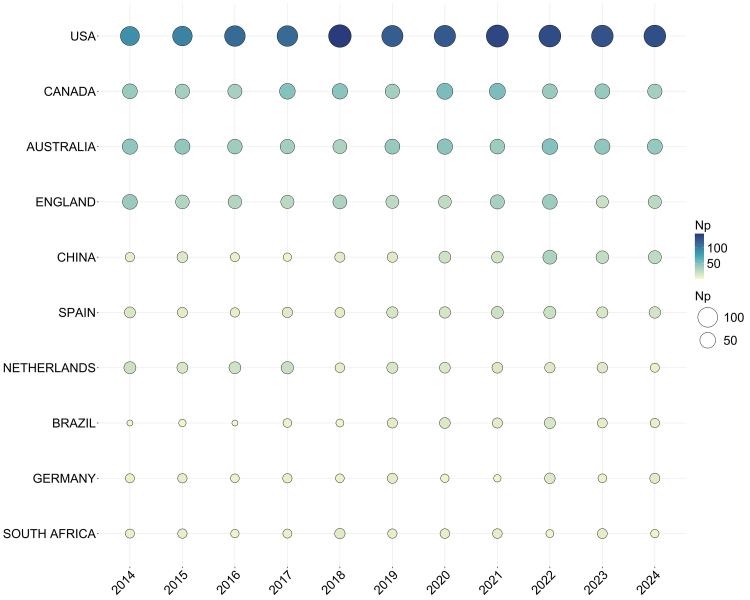
Top 10 countries in terms of annual publications on primary health care for chronic disease research, from 2014 to 2024. The circle’s size and colors show the number of papers. The larger the circle, the color from light yellow to blue, the higher the number of articles issued in that country. Nc = number of citations, Np = number of publications.

Furthermore, the annual publication trends from 2014 to 2024 (Fig. 3) show that the United States consistently produced the highest number of articles, peaking in 2018 with 146 publications. Since 2016, the United States has maintained a stable output of over 110 articles per year, underscoring its leading role in this research area. Canada and Australia also demonstrate consistent contributions, each publishing more than 30 articles annually over the past decade. Notably, China showed a significant increase in publications, with the highest output in 2022 (34 articles). Despite similar total publication volumes in the Netherlands, China, and Spain, the Netherlands and Spain had higher outputs in 2014, with 20 and 14 articles, respectively, compared with China’s 7 articles, indicating a later entry of China into this research field.

### 3.2. Annual trends in the quantity of paper publications

**Figure 4.**
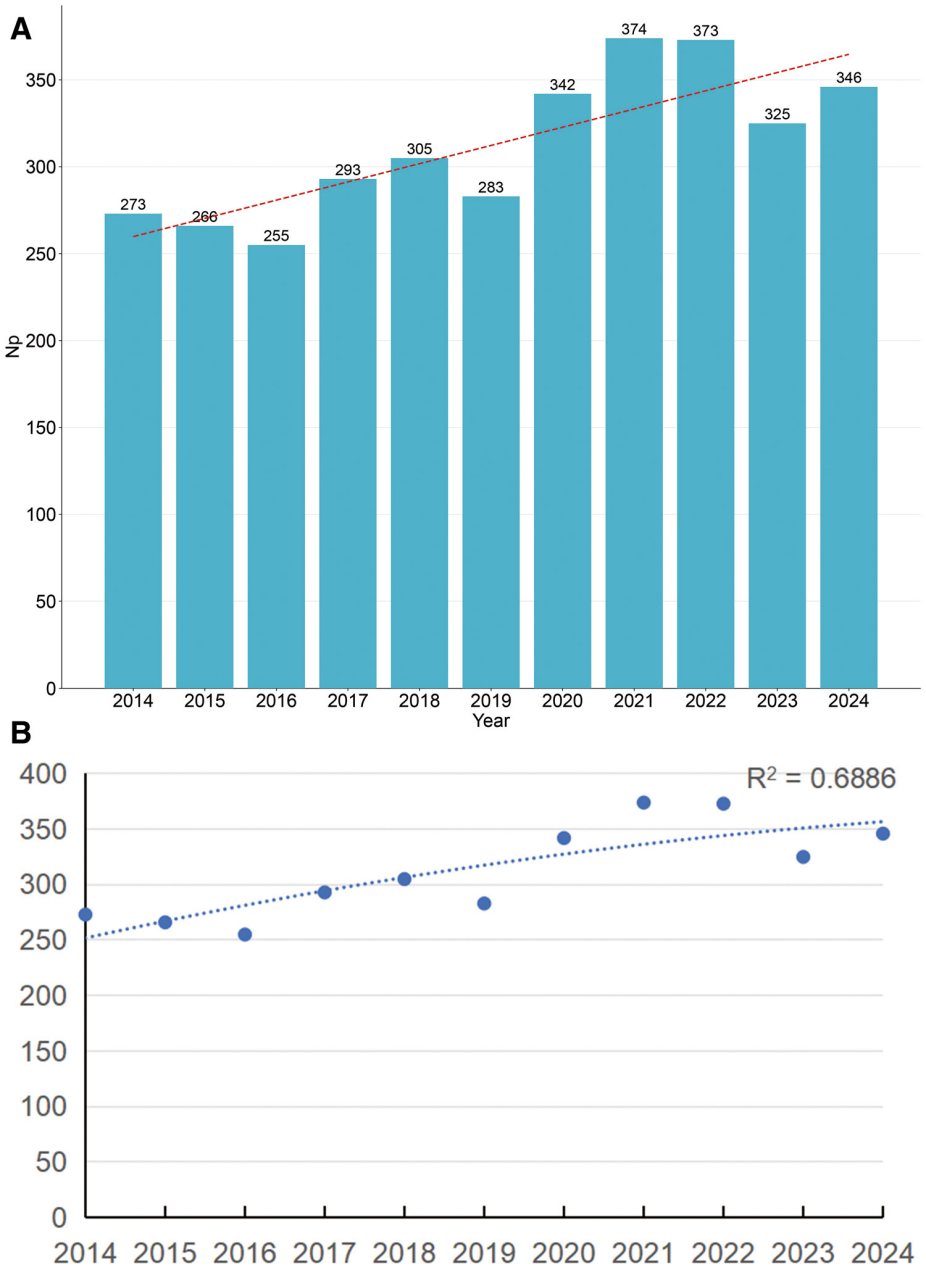
(A) The number of publications by year during the last 11 years. (B) Curve fitting of publications’ overall yearly growth trend.

The annual Np related to PHC for chronic disease shows a dynamic trend over the past decade. As illustrated in Figure 4A, the lowest publication count was in 2016, with 255 papers. This was followed by a gradual increase, peaking in 2021. Despite a decline in 2022 and 2023, the annual publication count remained above 300. Moreover, it rebounded to nearly 350 papers in 2024. The significant contribution of the United States heavily influences this overall trend, underscoring the country’s prolific output and its heightened focus on this research area.

The polynomial fitting curve of the annual publication trend, depicted in Figure 4B, further elucidates this trend. Although minor fluctuations occurred throughout the decade, the overall trajectory indicates an increase in publication volume. However, a slight downturn is noted in the recent 2 years, with a correlation coefficient (*R*^2^) of 0.6886, suggesting steady but slowed growth in research activity. These observations highlight the sustained and expanding interest in PHC for chronic disease, albeit with a recent moderation in growth.

### 3.3. Contributions of countries/regions to global publications

A detailed analysis of contributions by countries/regions, summarized in Table 2, reveals that the United States leads with 1344 publications, accounting for 39.13% of the total. Canada and Australia follow, with 483 (14.06%) and 475 (13.83%) publications, respectively. The United States also holds the highest citation count (Nc) at 23,752 citations, representing 43.13% of total citations, followed by Canada (11,756 citations) and Australia (9066 citations). The *H*-index rankings mirror these findings, with the United States at the top (*H*-index of 70), followed by England (56) and Canada (53). Despite the fact that the Np is higher in China than in the Netherlands and Spain, the Netherlands and Spain surpass China in both Nc and *H*-index, likely due to their earlier research initiatives and greater influence in these domains. Combining Np, Nc, and *H*-index metrics, the United States, Canada, Australia, and England consistently rank in the top 4, highlighting their leading roles in both publication volume and impact in PHC for chronic disease research.

### 3.4. Analysis of affiliations

Institutional contributions, as detailed in Table 3, indicate that the University of Toronto leads with 152 publications, closely followed by Harvard University with 150 publications. Among the top 10 institutions, the United States dominates with 7 institutions, while Australia, Canada, and England contribute one each. The top institutions by citation count include the University of Toronto, Harvard University, and the University of California System, with the latter 2 being US-based. Notably, the 4 institutions with the highest *H*-index are all from the United States, underscoring the country’s significant institutional impact on research in PHC for chronic disease.

**Table 3:** The top 10 most productive affiliations.

Rank	Affiliations	Country	Np	Nc	*H*-index
1	UNIVERSITY OF TORONTO	Canada	152	4040	31
2	HARVARD UNIVERSITY	USA	150	3941	36
3	UNIVERSITY OF CALIFORNIA SYSTEM	USA	131	2404	28
4	US DEPARTMENT OF VETERANS AFFAIRS	USA	131	3387	33
5	VETERANS HEALTH ADMINISTRATION VHA	USA	128	3352	33
6	HARVARD UNIVERSITY MEDICAL AFFILIATES	USA	125	3493	34
7	HARVARD MEDICAL SCHOOL	USA	89	2222	27
8	UNIVERSITY OF SYDNEY	Australia	88	1573	21
9	UNIVERSITY OF CALIFORNIA SAN FRANCISCO	USA	75	1423	23
10	UNIVERSITY OF LONDON	England	74	2898	30

Nc = number of citations, Np = number of publications.

### 3.5. Analysis of authors

Table 4 presents the 10 most prolific authors in the field of PHC for chronic disease, collectively contributing 194 publications, accounting for 5.65% of all submitted articles. Among these authors, Fortin Martin (25 publications), Hudon Catherine (18 publications), and Poitras Marie-Eve (16 publications) are affiliated with the University of Sherbrooke, Canada. The distribution includes 7 authors from Canada, 2 from the United States, and 1 from Australia, indicating a concentration of leading researchers from these countries in this domain. Notably, Grunfeld, E from the University of Toronto, which is listed in Table 3 as the first of the most productive affiliations, underscores their significant contributions to research in PHC for chronic disease.

**Table 4:** The top 10 authors with the most publications.

Rank	Author	Affiliations	Country	Np	Nc	*H*-index
1	Halcomb, Elizabeth	University of Wollongong	Australia	25	421	13
2	Fortin, Martin	University of Sherbrooke	Canada	25	1421	15
3	Chouinard, Maud-Christine	Universite de Montreal	Canada	21	351	12
4	Bayliss, Elizabeth A.	University of Colorado	USA	19	626	13
5	Manca, Donna P	University of Alberta	Canada	19	281	8
6	Hudon, Catherine	University of Sherbrooke	Canada	18	325	12
7	Westfall, John Mark Jack	University of Colorado	USA	18	490	10
8	Grunfeld, E	University of Toronto	Canada	17	116	7
9	Poitras, Marie-Eve	University of Sherbrooke	Canada	16	337	10
10	Aubrey-Bassler, Kris	Mem Univ Newfoundland	Canada	16	804	9

Nc = number of citations, Np = number of publications.

### 3.6. Analysis of journals

As shown in Table 5, the top 10 journals publishing research on PHC for chronic disease have a journal impact factor (JIF) ranging from 1.2 to 4.6, with an average of 3.16. BMJ Open ranks highest in terms of the Np. BMC Health Services Research and BMC Family Practice rank highest in terms of both the Nc and *H*-index. BMJ Open is a medical journal focusing on clinical medicine, public health, and epidemiology, with an emphasis on patient- and clinician-relevant research. BMC Health Services Research publishes articles on all aspects of health services research, while BMC Family Practice particularly relates to PHC research.

**Table 5:** The top 10 most-published journals.

Rank	Journal	Np	Nc	*H*-index	JIF (2023)	JCI (2023)
1	BMJ OPEN	139	1931	22	2.4	0.68
2	BMC HEALTH SERVICES RESEARCH	120	2415	27	2.7	0.92
3	BMC FAMILY PRACTICE	94	3267	27	3.2	1.44
4	FAMILY PRACTICE	75	1157	19	2.4	1.05
5	PLOS ONE	75	2321	23	2.9	0.88
6	JOURNAL OF THE AMERICAN BOARD OF FAMILY MEDICINE	70	909	18	2.4	1.36
7	JOURNAL OF GENERAL INTERNAL MEDICINE	69	1680	23	4.3	1.34
8	BMC PRIMARY CARE	53	262	10	2	1.22
9	INTERNATIONAL JOURNAL OF ENVIRONMENTAL RESEARCH AND PUBLIC HEALTH	50	609	14	4.6	0.93
10	AUSTRALIAN JOURNAL OF PRIMARY HEALTH	47	394	13	1.2	0.49
10	BMC PUBLIC HEALTH	47	1392	15	3.5	1.1

JCI = journal citation indicator, JIF = journal impact factor, Nc = number of citations, Np = number of publications.

The International Journal of Environmental Research and Public Health, with a JIF (2023) of 4.6, focuses on the interactions between environmental quality and health, reflecting a broad interest in the societal and cultural factors influencing health outcomes. The Journal of General Internal Medicine, with a JIF (2023) of 4.3, is dedicated to advancing clinical research to improve patient care, primary care, general internal medicine, and hospital medicine education. BMC PUBLIC HEALTH, with a JIF (2023) of 3.5, is dedicated to public health and related interdisciplinary disciplines.

The substantial publication volume and high quality of articles in these journals underscore their influence and relevance in PHC for chronic disease research.

### 3.7. Publications in top-tier journals

Table 6 details the publications on PHC for chronic disease in top-tier journals such as CA-A CANCER JOURNAL FOR CLINICIANS, BMJ-BRITISH MEDICAL JOURNAL, and NATURE REVIEWS DISEASE PRIMERS, alongside other high-IF journals. The top 10 journals by IF collectively account for 27 publications. The United States leads with 16 articles published in these high-impact journals, including one in CA-A CANCER JOURNAL FOR CLINICIANS with a JIF (2023) of 521.6. China and Canada follow with 4 and 3 publications each. This distribution reflects the significant emphasis and strong research foundations these countries have in the field of PHC for chronic disease.

**Table 6:** Correlation of high-quality journals with high attention and the most productive countries.

Rank	Journal	Np (Total)	JIF (2023)	Np						
				USA	Canada	China	England	New Zealand	Denmark	Scotland
1	CA-A CANCER JOURNAL FOR CLINICIANS	1	521.6	1						
2	BMJ-BRITISH MEDICAL JOURNAL	5	93.7	3		1	1			
3	NATURE REVIEWS DISEASE PRIMERS	2	79					1	1	
4	JAMA-JOURNAL OF THE AMERICAN MEDICAL ASSOCIATION	5	63.5	4	1					
5	NATURE REVIEWS NEUROLOGY	1	28.2	1						
6	LANCET PUBLIC HEALTH	3	25.4			2				1
7	PROCEEDINGS OF THE IEEE	1	23.2	1						
8	JAMA INTERNAL MEDICINE	7	22.3	5	2					
9	JOURNAL OF THE AMERICAN COLLEGE OF CARDIOLOGY	1	21.7			1				
10	ANNUAL REVIEW OF PUBLIC HEALTH	1	21.4	1						

JIF = journal impact factor, Np = number of publications.

### 3.8. Analysis of highly cited articles

Highly cited articles within the field of PHC for chronic disease demonstrate the breadth and depth of research contributions. As shown in Table 7, 2 articles have been cited over 600 times, and another 2 have been published in journals boasting an IF of 79. The article titled “Gout (primer)” published in “NATURE REVIEWS DISEASE PRIMERS” (IF = 79) has garnered significant attention, with 394 citations. This article delves into the pathogenesis of hyperuricemia, clinical advancements in the management of gout, and best practices for patient care, emphasizing the use of anti-inflammatory medications and urate-lowering therapy. Another one titled “Multimorbidity” with 364 citations analyzes the underlying mechanisms for the development of multimorbidity, and explores the interventions for multimorbidity to reconfigure the medical support management of multimorbidity.

**Table 7:** The top 10 highest-cited articles.

Rank	Article	JIF (2023)	Total citation	Type of study
1	De Onis M, Branca F. Childhood stunting: a global perspective[J]. Maternal & child nutrition, 2016, 12: 12-26.	2.8	665	Review
2	Thompson A E, Anisimowicz Y, Miedema B, et al. The influence of gender and other patient characteristics on health care-seeking behaviour: a QUALICOPC study[J]. BMC family practice, 2016, 17: 1-7.	3.2	609	Article
3	Smith S M, Wallace E, O’Dowd T, et al. Interventions for improving outcomes in patients with multimorbidity in primary care and community settings[J]. Cochrane Database of Systematic Reviews, 2016 (3).	8.8	478	Review
4	Garvey W T, Mechanick J I, Brett E M, et al. American Association of Clinical Endocrinologists and American College of Endocrinology comprehensive clinical practice guidelines for medical care of patients with obesity[J]. Endocrine Practice, 2016, 22: 1-203.	3.7	436	Article
5	Stuebe A, Auguste T, Gulati M. Optimizing postpartum care[J]. Obstetrics and Gynecology, 2018, 131(5): E140-E150.	5.8	409	Article
6	Koné Pefoyo A J, Bronskill S E, Gruneir A, et al. The increasing burden and complexity of multimorbidity[J]. BMC public health, 2015, 15: 1-11.	3.5	406	Article
7	Dalbeth N, Choi H K, Joosten L A B, et al. Gout (primer)[J]. Nature Reviews: Disease Primers, 2019, 5(1).	79	394	Article
8	Skou S T, Mair F S, Fortin M, et al. Multimorbidity[J]. Nature Reviews Disease Primers, 2022, 8(1): 48.	79	364	Article
9	Bähler C, Huber C A, Brüngger B, et al. Multimorbidity, health care utilization and costs in an elderly community-dwelling population: a claims data based observational study[J]. BMC health services research, 2015, 15: 1-12.	2.7	320	Article
10	McPhail S M. Multimorbidity in chronic disease: impact on health care resources and costs[J]. Risk management and healthcare policy, 2016: 143-156.	2.7	307	Review

JIF = journal impact factor.

Furthermore, systematic reviews published in the “Cochrane Database of Systematic Reviews” are prominent in this list. Smith S M et al’s review on interventions for multimorbidity in primary care (478 citations) highlights the effectiveness of comprehensive care approaches and supports policy implementations for managing multimorbidity. These articles collectively underscore the significance of multidisciplinary and patient-centered care models in PHC settings.

### 3.9. GCS of key publications

**Figure 5.**
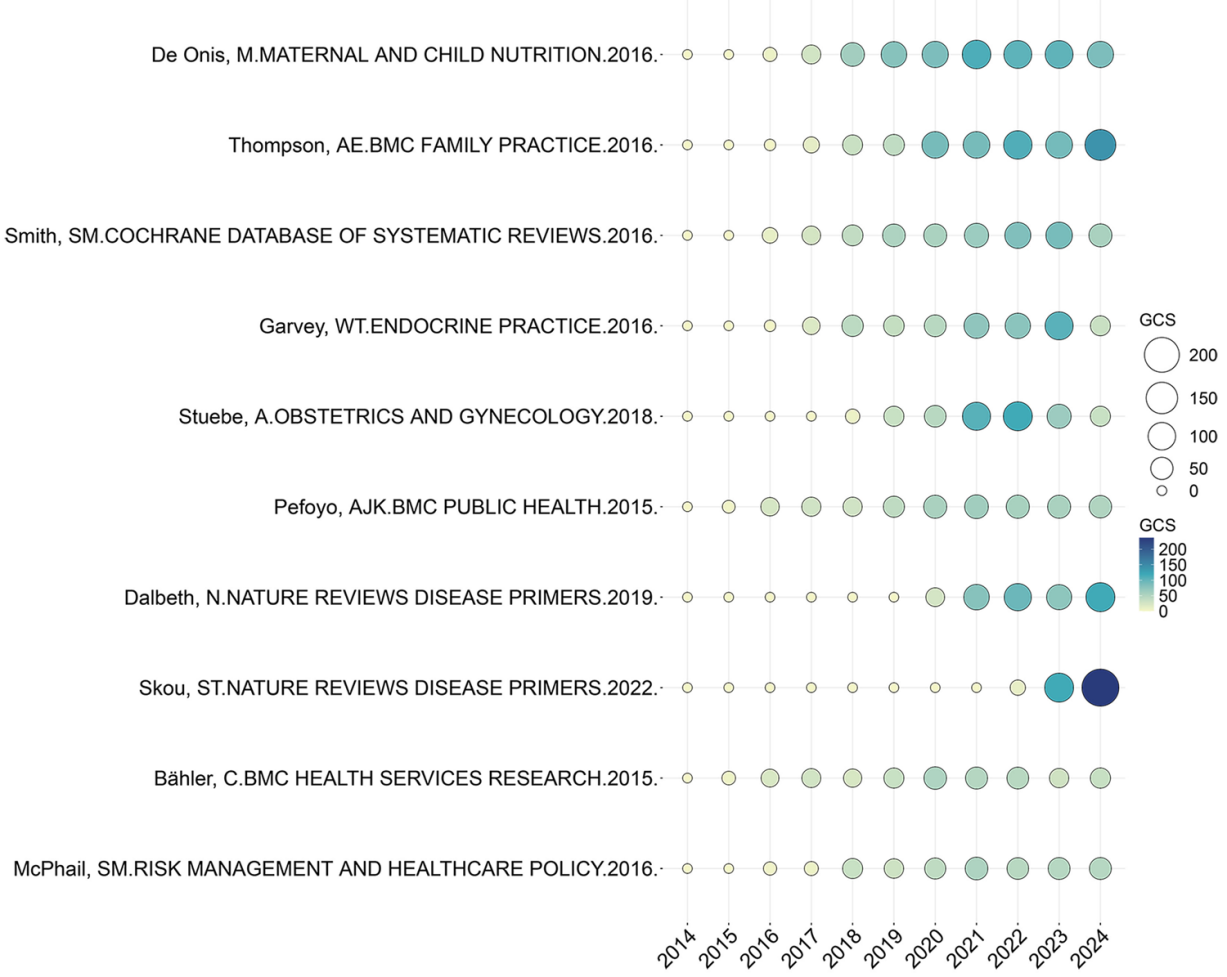
The yearly number of global citations for papers having a high GCS. The circle’s size and colors show the GCS of papers. The larger the circle and the color from light yellow to blue, the higher the GCS of the article and the more influential it is in the field. GCS = global citation score.

The annual global citation counts for high-impact papers reveal a concentrated citation peak in recent years: annual peaks in citation scores, particularly for Skou S T et al’s research on the prevalence, determinants, and patterns of multimorbidity, with a GCS of 237 in 2024 (Fig. 4). Stuebe A’s research on comprehensive postpartum care in 2022 (GCS = 117) illustrates that the evolving focus on chronic disease management and the expanding scope of PHC. De Onis M’s study on linear growth failure and Thompson A E’s patient-reported outcomes on PHC utilization further enrich the understanding of PHC for chronic diseases. These studies highlight the diverse populations and varied health concerns addressed within this research domain, emphasizing the need for tailored care strategies (Fig. 5).

**Figure 6.**
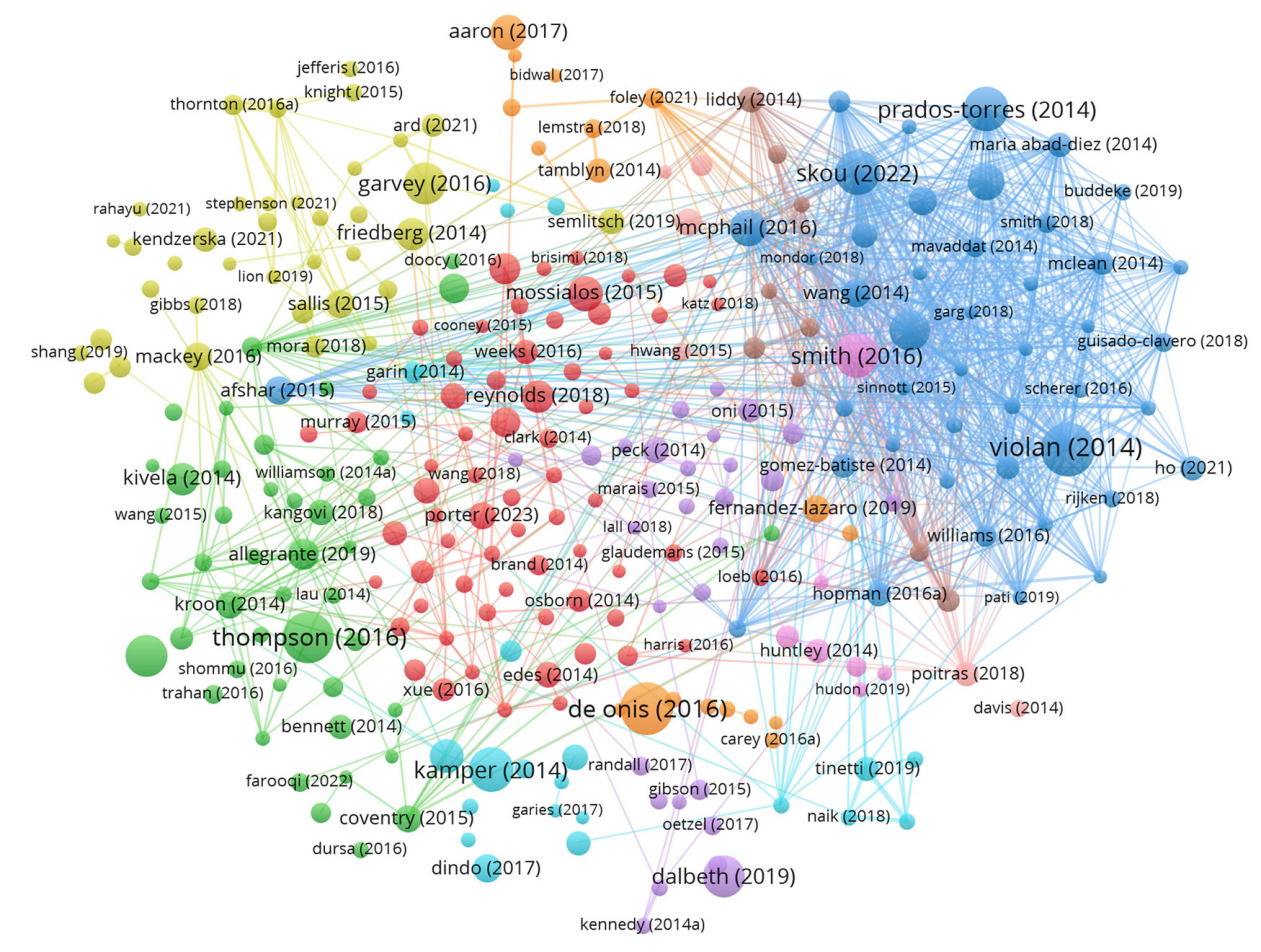
Network of document citation. Given a large number of references available, the minimum number of citations for a reference was placed at 50. Of the 3435 papers, 296 were chosen for citation analysis. The different colors of the nodes represent different documents, with larger nodes meaning more frequently cited articles.

Figure 6 shows the 296 most frequently cited articles (other publications with over 50 citations), corresponding to Figure 5, where articles with higher GCS have larger nodes and occupy the core positions of the grid.

**Figure 7.**
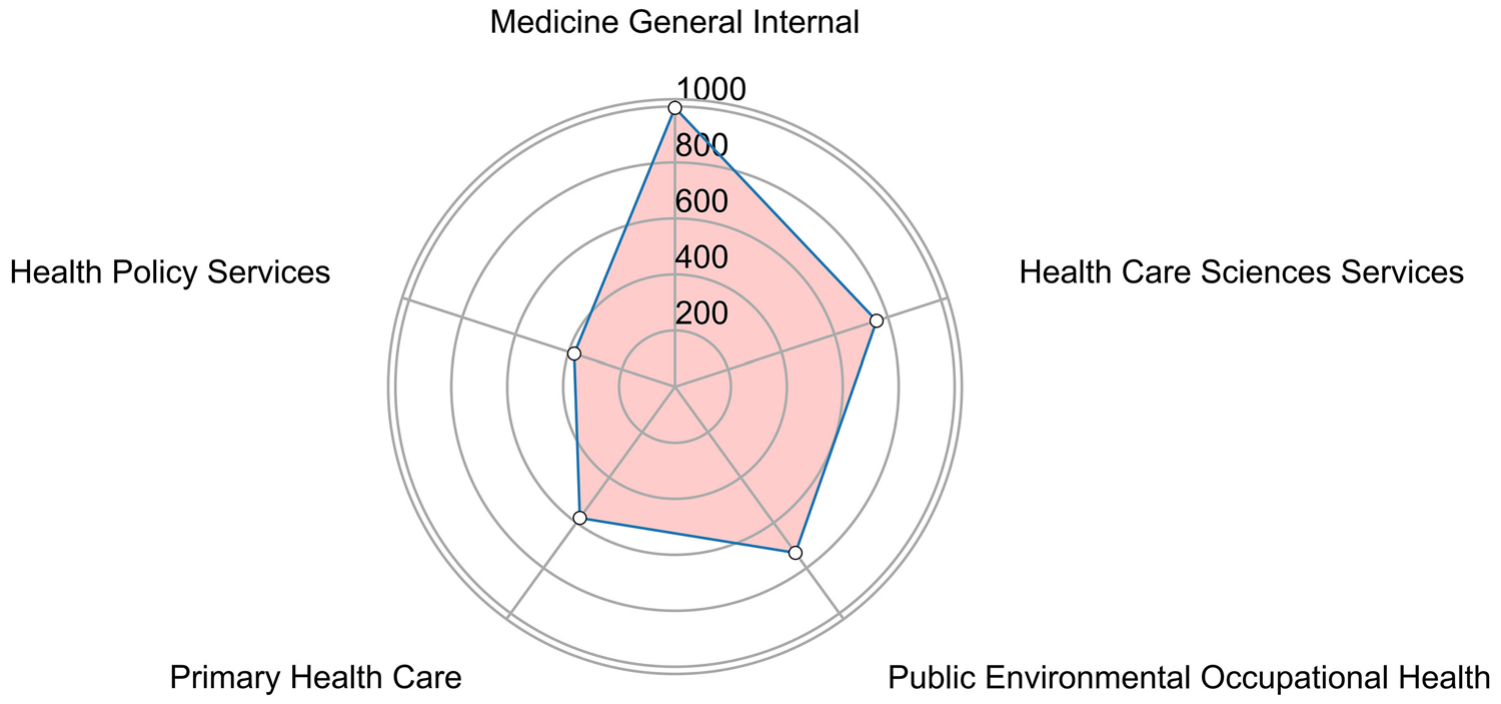
Radar map of the top 5 research-productive categories in primary health care for chronic disease research.

Research within the PHC domain spans several key science categories. “Medicine General Internal” emerges as the most prevalent category with 995 articles, reflecting the broad applicability of PHC principles across internal medicine. Other significant categories include “Health Care Sciences Services,” “Public Environmental Occupational Health,” “Primary Health Care,” and “Health Policy Services,” each contributing to the multidisciplinary nature of PHC research (Fig. 7).

### 3.10. Analysis of co-cited references

**Figure 8.**
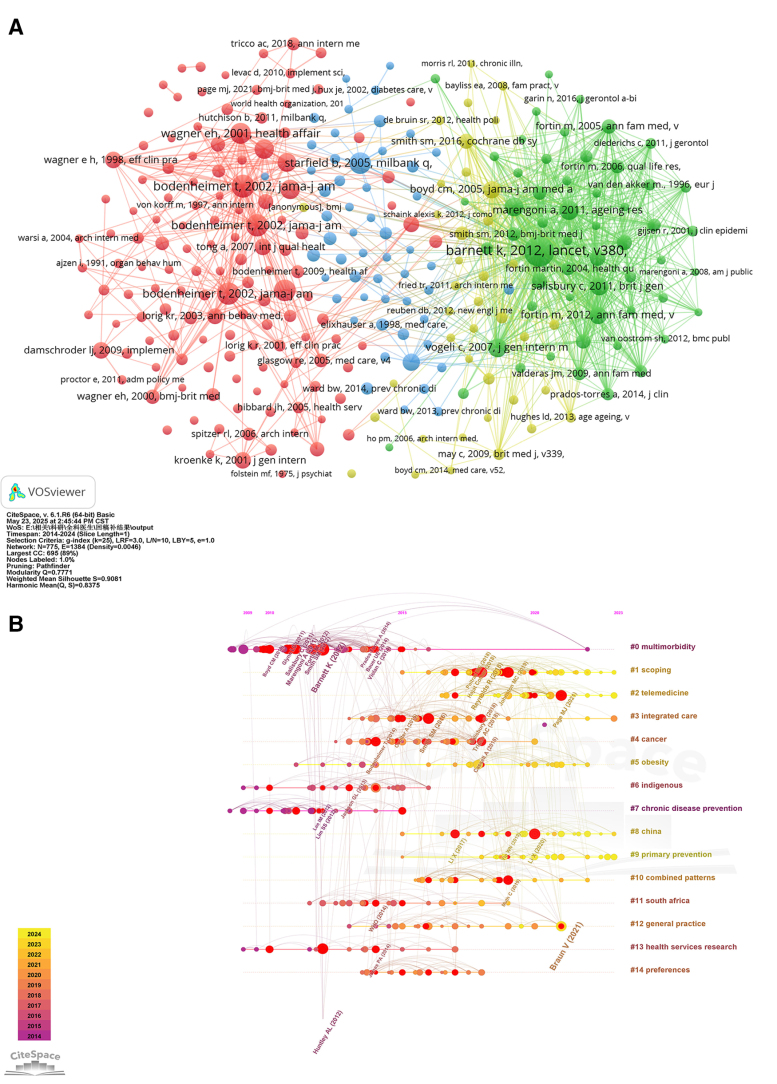
Mapping based on co-cited references from primary health care for chronic disease research. (A) A network diagram of co-cited references. Of the 109,566 references, 281 (divided into 4 clusters) were cited at least 15 times. (B) The timeline distribution of the top 15 clusters’ timeline distribution.

Co-citation analysis, as opposed to global citation analysis, focuses on research topics closely related to a specific field. Due to the large volume of references, a minimum citation threshold of 15 times per document was set. From the 109,566 retrieved documents, 281 were selected for co-citation analysis. Connections between nodes represent co-citation in the same publication, with shorter lines indicating closer associations. Different node colors were used to categorize papers into clusters (Fig. 8A).

**Figure 9.**
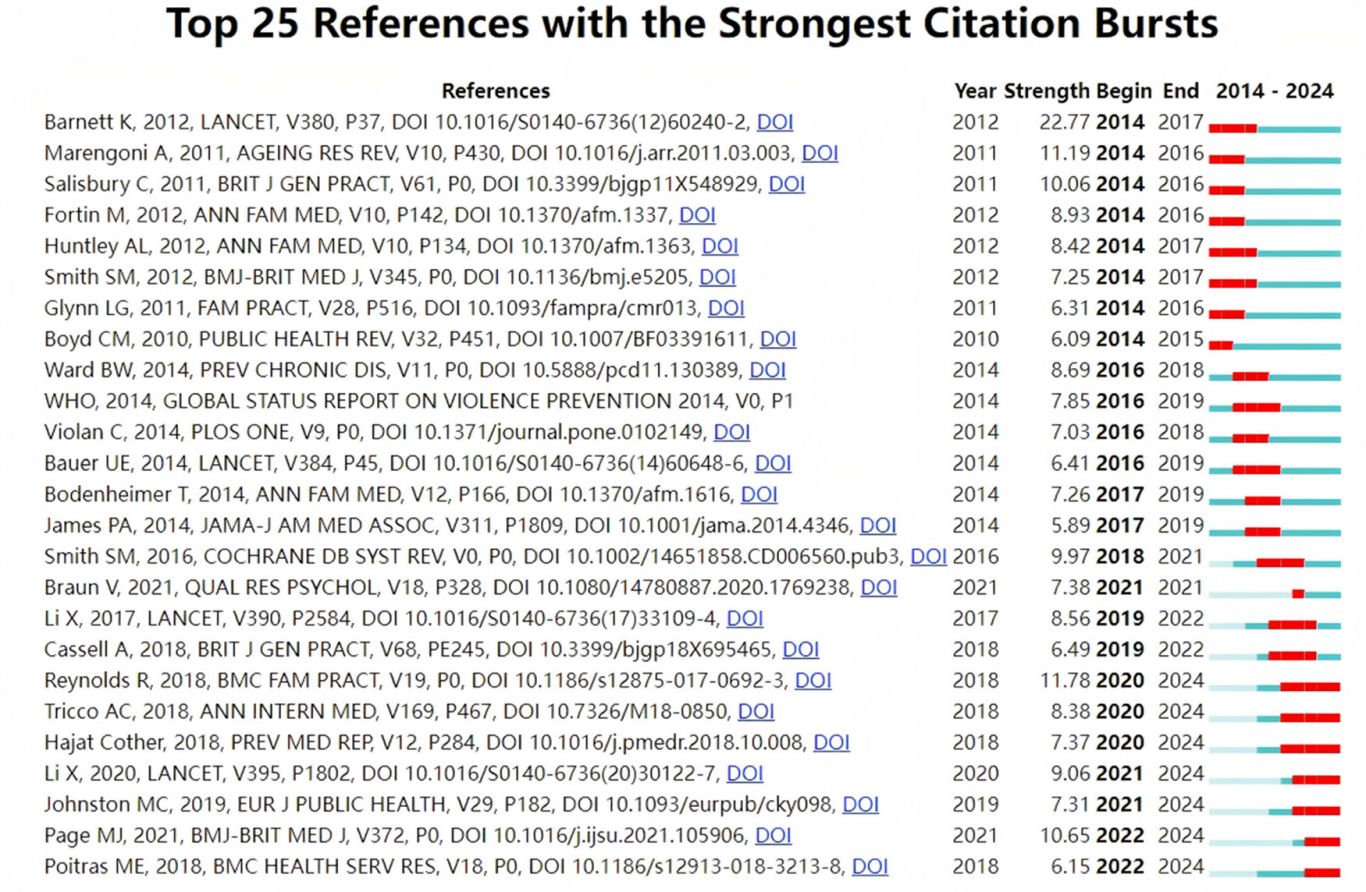
The top 25 co-cited references with the most citation burstiness. The years between “Begin” and “End” represent the period when the reference was more influential. Years in light green mean that the reference has not yet appeared, years in dark green mean that the reference is less influential, and years in red mean that the reference is more influential.

Cluster 1 (red) includes 133 papers, emphasizing the current status of PHC for chronic disease. Cluster 2 (green) comprises 55 papers, primarily focusing on the epidemiology and impact of chronic disease within PHC. Cluster 3 (blue) includes 54 papers, centered on the utilization of PHC for chronic disease. Cluster 4 (yellow) consists of 39 papers, concentrating on various management models of PHC for chronic disease. This clustering reveals that most research is concentrated on the status, impact, and utilization of PHC for chronic disease. Figure 8B illustrates the most significant references in terms of burst length, burst strength, and burst time. The top 15 clusters of co-cited references include “multimorbidity,” “scoping,” “telemedicine,” “integrated care,” “cancer,” “obesity,” “indigenous,” “chronic disease prevention,” “China,” “primary prevention,” “combined patterns,” “South Africa,” “general practice,” “health services research,” and “preferences.” Figure 9 depicts the top 25 references with the most robust citation bursts. The study by Barnett K et al shows the highest strength (22.77), challenging the single-disease framework prevalent in healthcare, medical research, and education, advocating for comprehensive, personalized care by GPs, especially in socioeconomically deprived areas. This study’s citation burst lasted until 2017.

Following closely is the research by Reynolds R et al., with a burst strength of 11.78, which is a systematic review of chronic disease management interventions in primary care, with a citation burst extending to 2024. Marengoni A et al., with a burst strength of 11.19, is also a systematic review of the existing scientific evidence of the occurrence, causes, consequences, nursing mode, and nursing quality of multimorbidity, but the population is elderly, with a citation burst extending to 2016. The research by Page MJ et al., with a burst strength of 10.65, introduces the Preferred Reporting Items for Systematic Reviews and Meta-Analyses (PRISMA) 2020 statement – a revised guideline for systematic review reporting, applicable to the PHC for chronic disease domain, with a citation burst extending to 2024. The research by Salisbury C et al., with a burst strength of 10.06, is a retrospective cohort study about the epidemiology and impact of multimorbidity in primary care, with a citation burst extending to 2016. Among these 25 references, 7 have a citation burst ending in 2024, all of which are reviews in the PHC for the chronic disease field. Notable studies include systematic reviews by Tricco AC et al and Page MJ et al on PRISMA updates, Reynolds R et al on chronic disease management interventions in primary care, and Li X et al on challenges and recommendations for PHC quality in China. The remaining reviews focus on definitions, current status, and optimal clinical management evidence for multiple chronic conditions.

### 3.11. Keyword analysis from VOSviewer and CiteSpace

**Figure 10.**
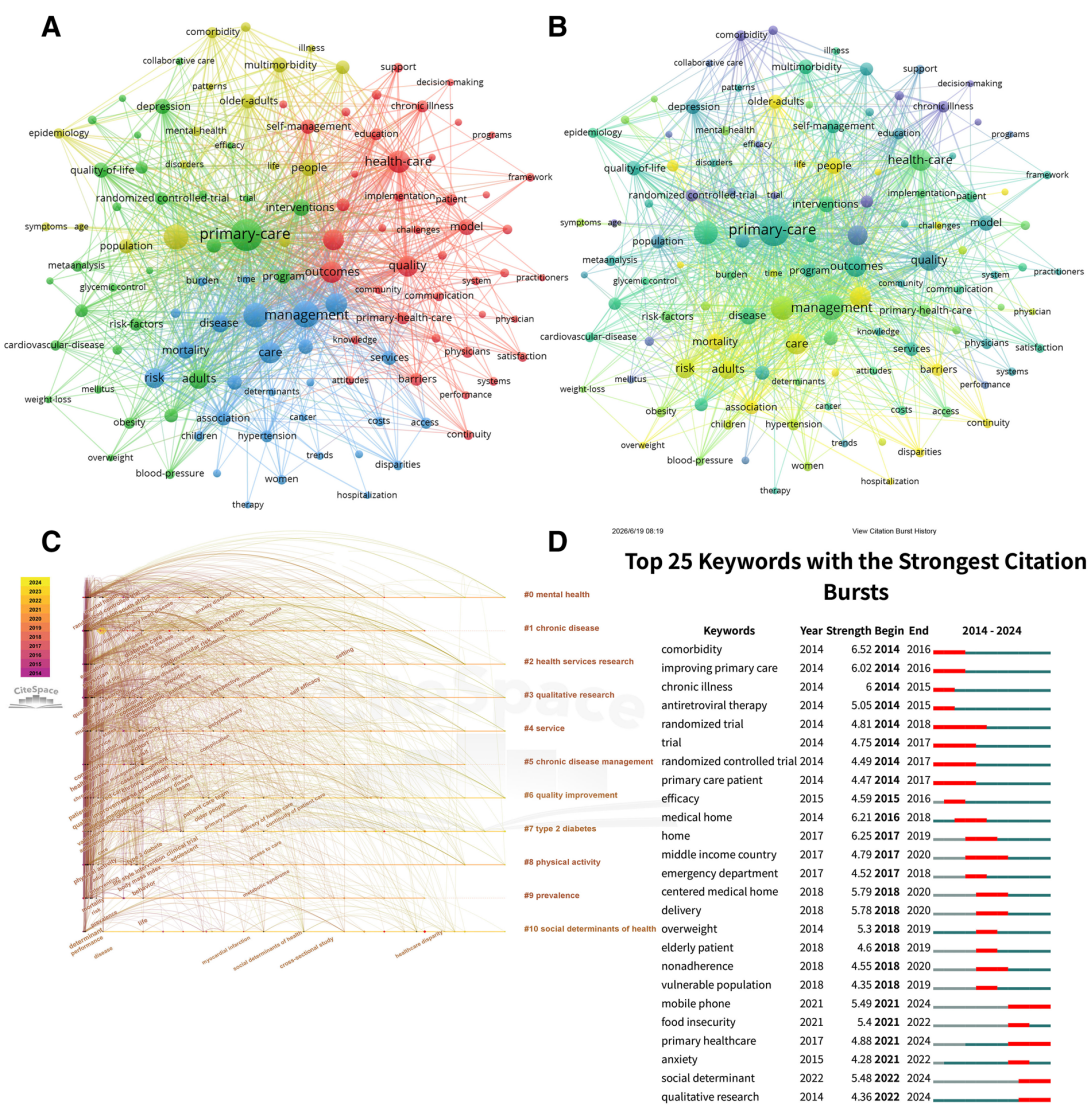
Keyword mapping of primary health care for chronic disease research. (A) Using different colors, the 119 terms that appeared more than 30 times were separated into 4 clusters: Group 1 (red) focuses primarily on researching the management mode of chronic diseases; group 2 (green) mainly emphasizes health and disease risks; group 3 (blue) concentrates on the effectiveness of primary care; and group 4 (yellow) primarily addresses the current epidemiology of chronic diseases. The size of the nodes indicates occurrence frequency. (B) Keyword visualization according to the average publication year. The different colors indicate the relevant year of publication. Yellow keywords came later than purple keywords. (C) Timeline distribution of keyword cluster analysis. (D) The top 25 keywords with the most bursts. The years between “Begin” and “End” represent the period when the keyword was more influential. Years in light green mean that the keyword has not yet appeared, years in dark green mean that the keyword is less influential, and years in red mean that the keyword is more influential.

VOSviewer and CiteSpace evaluated keywords collected from the titles and abstracts of 3435 publications. As shown in Figure 10A, cluster 1 (red) primarily focuses on research on chronic disease management models, cluster 2 (green) on health and disease risks, cluster 3 (blue) on the efficacy of primary care, and cluster 4 (yellow) on the epidemiological status of chronic diseases. The most frequently occurring keywords include “primary care,” “management,” “health-care,” “outcomes,” “quality,” “mortality,” and “multimorbidity,” indicating a strong research focus on the epidemiology and management of chronic diseases within primary care settings.

Figure 10B categorizes all keywords by average publication year (APY). The latest keywords include “medication adherence” (cluster 2, APY: 2020.18), “association” (cluster 3, APY: 2020.08), “perspectives” (cluster 1, APY: 2020.03), “telemedicine” (cluster 1, APY: 2020.03), and “life” (cluster 4, APY: 2020), still revolving around the epidemiology and efficacy of chronic disease management in primary care.

In addition, keywords such as “mortality” (cluster 3, APY: 2019.83) and “hospitalization” (cluster 3, APY: 2019.59) remain hot topics, highlighting the ongoing interest in the efficacy of primary care for chronic diseases. Keywords such as “chronic diseases” (cluster 4, APY: 2017.75), “comorbidity” (cluster 4, APY: 2017.32), “multiple chronic conditions” (cluster 4, APY: 2018.55), and “epidemiology” (cluster 4, APY: 2018.84) remain hot topics, highlighting the ongoing interest in the epidemiological aspects of chronic diseases.

“Mental health,” “chronic disease,” “health services research,” “qualitative research,” “service,” “chronic disease management,” “quality improvement,” “type 2 diabetes,” “physical activity,” “prevalence,” and “social determinants of health” have consistently been focal points in PHC research for chronic disease (Fig. 10C).

Meanwhile, Figure 10D shows that “mobile phone” and “primary healthcare” are the most popular keywords from 2021 to the present, and “social determinant” and “qualitative research” are the most popular keywords from 2022 to the present. The high strength of “comorbidity,” “home,” “medical home,” and “improving primary care” indicates heightened concern for these conditions in recent years. “Improving Primary Care” highlights the urgency of improving primary care, while “mobile phones” proposes concrete measures and reinforces the growing importance of smartphones in health monitoring and management. The emphasis on “social determinants of health” and “primary healthcare” highlights the significant impact of social factors on health, advocating for enhanced community involvement in primary healthcare. “Qualitative research” as a prevalent keyword suggests a predominant trend towards qualitative studies in the PHC for the chronic disease field.

The main theme across Figure 10A to D is the study of the epidemiological status of chronic diseases and the effectiveness of primary care management models. Future research is likely to increasingly explore new primary care management models, leveraging internet technologies to enhance health management efficiency, reduce the incidence of sub-health and chronic diseases, and improve treatment and management outcomes for patients with multiple conditions, thereby enhancing health-related quality of life.

## 4. Discussion

This study represents the first bibliometric analysis of research on PHC for chronic diseases. Utilizing the WoSCC, we aimed to analyze the development trends and research hotspots in this field. We retrieved 3435 articles and reviews published between 2014 and 2024. Despite minor fluctuations in the number of published papers over this decade, polynomial fitting curves indicate an overall increasing trend, with a slight decline in 2022 and 2023, but a rebound to high levels by 2024. This suggests a growing interest among researchers in PHC for chronic diseases. The distribution of publications is global, yet significant disparities in productivity exist across different regions. As shown in Figure 2 and Table 2, the United States leads with the highest Np in this field, followed by Canada. However, the publication ratio between the United States and Canada is nearly 2.78:1, indicating the United States’ dominance. The top 10 institutions in this field are predominantly from the United States (7 institutions); the remaining 3 are from Australia, Canada, and England. Among the top 10 prolific scholars, 7 are from Canada, 2 are from the United States, and 1 is from Australia. This highlights the prominence of American institutions and the substantial contributions from Australia and Canada, not only through institutions but also via prolific scholars, explaining these countries’ significant influence over the past decade. Table 2 illustrates that the United States holds the highest *H*-index (70), followed by Canada (53) and England (56). Evaluating Np, Nc, and H-index collectively, the United States, Canada, Australia, and England consistently rank in the top 4, indicating their high productivity and significant impact in this field. Canada notably hosts the leading research institution, the University of Toronto, and the most prolific scholar, Fortin Martin, from the University of Sherbrooke. Three of the top 10 prolific scholars are affiliated with the University of Sherbrooke, underscoring Canada’s dedication to this research domain.

Although the Np value of China is higher than that of Spain and the Netherlands, both the Nc and h indexes of the Netherlands and Spain exceed those of China, which may be due to the fact that these 2 countries started early and had a large influence in this field. In 1970, Nivel at the Netherlands Institute for Health Services Research launched a paper-based primary care sentinel network to investigate the epidemiology of certain diseases and inform GPs. By the 1990s, the network transitioned from paper-based data collection to extracting data from electronic health records and expanded to include not only GPs but also other primary healthcare providers. This network is now known as the Nivel Primary Care Database.^[18]^ In Spain, primary care serves as the first level of care, providing comprehensive services including health promotion, chronic disease prevention, and social assistance, while hospital care represents the second level, offering the most complex and expensive diagnostic and treatment resources. For chronic diseases, primary care utilization is higher than that of hospital care.^[19]^

Among the top 10 most-published journals in this field, 6 have Nc values exceeding 1000, and 5 have *H*-index values of 20 or higher. The JIF for 2023 ranges from 1.2 to 4.6, and the journal citation indicator for 2023 ranges from 0.49 to 1.36, indicating the substantial impact and quality of publications in this field. The top 3 countries publishing high-quality research papers are the United States (16 papers), China (4 papers), and Canada (3 papers), demonstrating the high research quality in these nations.

Annual peaks in citation scores, particularly for Skou S T et al’s research on the prevalence, determinants, and patterns of multimorbidity, with a GCS of 237 in 2024.^[20]^ This paper was published in 2022,^[21]^ which peaked in GCS in 2024, reflecting its growing recognition and importance. Significantly cited papers include the work by Dalbeth N and colleagues on PHC for gout, which discusses best practices in gout management and the widespread use of anti-inflammatory drugs for acute attacks and urate-lowering therapy for long-term management.^[21]^ This paper was published in 2019,^[21]^ which peaked in GCS in 2024, reflecting its growing recognition and importance.

In the past 5 years, GCS has primarily focused on the research conducted by De Onis M and Thompson A E, among others. De Onis M’s research suggests that PHC institutions should not only focus on the multiple diseases of the elderly but also shift their attention to linear growth failure in children, thereby reducing the risk of chronic diseases in adulthood.^[22]^ Thompson A E’s research emphasizes the utilization of primary care by different genders.^[23]^ In summary, research on PHC for chronic disease continues to attract significant attention from many researchers. The GCS studies by Stuebe A and colleagues reached their peak in 2022, concentrating on comprehensive postpartum follow-up, especially for mothers with chronic diseases such as hypertension, obesity, diabetes, thyroid diseases, kidney diseases, and mood disorders. They proposed a new model of postpartum care, expanding the breadth of PHC and optimizing family care and support postpartum.^[24]^ Evidently, the field of PHC for chronic disease encompasses various aspects, different research populations, and different research focuses, demonstrating both breadth and depth. Figure 6 shows the 296 most frequently cited articles (other publications with over 50 citations), corresponding to Figure 5, where articles with higher GCS have larger nodes and occupy the core positions of the grid. The most popular research category is Medicine General Internal (995 articles), followed by Health Care Sciences Services (758 articles), Public Environmental Occupational Health (733 articles), PHC (579 articles), and Health Policy Services (379 articles).

The co-citation network results indicate that research on PHC for chronic disease mainly revolves around 4 clusters: the current status of PHC for chronic disease; epidemiology and impact of chronic disease on PHC; the utilization of PHC for chronic disease; and various models of management for PHC for chronic disease. Based on these clusters, we find that most studies focus on the current status, impact, and utilization of PHC for chronic disease. From the timeline views of co-cited literature and keywords (Figs. 8B and 10C), it is evident that the enduring hot topics include “multimorbidity,” “Chronic illness,” and “Primary health care.” Analyzing the top 25 References with the Strongest Citation Bursts shows that the research by Barnett K et al.^[25]^ has the highest strength. Their findings challenge the single-disease framework prevalent in most healthcare, medical research, and medical education, proposing the need for a supplementary strategy to support primary care clinicians in providing personalized, comprehensive, and continuous care, especially in socioeconomically deprived areas.^[25]^

Following closely is the research by Reynolds R et al., with a burst strength of 11.78, which is a systematic review of chronic disease management interventions in primary care, with a citation burst extending to 2024.^[26]^ Marengoni A et al., with a burst strength of 11.19, is also a systematic review of the existing scientific evidence of the occurrence, causes, consequences, nursing mode and nursing quality of multimorbidity, but the population is elderly, with a citation burst extending to 2016.^[27]^ The research by Page MJ et al,^[28]^ with a strength of 10.68, introduces the PRISMA 2020 statement, a new guideline for systematic review reporting. This guideline includes an expanded checklist detailing reporting recommendations for each item, the PRISMA 2020 abstract checklist, and revised flow diagrams for original and updated reviews. This study can be used to guide systematic reviews and meta-analyses in the field of PHC for chronic disease, with a cutoff year of 2024. The research by Salisbury C et al., with a burst strength of 10.06, is a retrospective cohort study about the epidemiology and impact of multimorbidity in primary care, with a citation burst extending to 2016.^[29]^ Among these 25 references, 7 have a cutoff year of 2024, all of which are reviews in the field of PHC for chronic disease, mainly focusing on the current status of PHC for multiple chronic diseases and exploring the best clinical management evidence.^[26,30–34]^

Garin N et al analyzed data from the European Aging Cooperation Research Project (Finland, Poland, and Spain) and the World Health Organization’s Study on Global Aging and Adult Health (China, Ghana, India, Mexico, Russia, and South Africa). Their results indicate that the overall prevalence of multimorbidity is high across these countries, with hypertension, cataracts, and arthritis being the most common comorbid conditions. Each country exhibited 2 or 3 distinct multimorbidity patterns, such as “cardiovascular-respiratory” (angina, asthma, and COPD), “metabolic” (diabetes, obesity, and hypertension), and “mental-arthritis” (arthritis and depression).^[10]^ The presence of specific multimorbidity patterns in some countries suggests the existence of common underlying pathogenic factors. It is crucial to focus on researching these patterns to develop more targeted preventive measures and create new integrated approaches to manage these co-occurring conditions.

Using VOSviewer and CiteSpace, keywords were evaluated from the titles and abstracts of 3435 publications. As shown in Figure 10A, group 1 (red) mainly focuses on chronic disease management models, group 2 (green) on health and disease risks, group 3 (blue) on primary care effectiveness, and group 4 (yellow) on the current epidemiology of chronic diseases. The most frequently appearing keywords were “primary care,” “management,” “health-care,” “outcomes,” “quality,” “mortality,” and “multimorbidity,” indicating that research related to PHC for chronic diseases primarily concentrates on the epidemiology and management of chronic diseases within primary care settings.

As depicted in Figure 10B, the most recent keywords are “medication adherence,” “association,” “perspectives,” “telemedicine” and “life,” which still revolve around the epidemiology and efficacy of chronic disease management in primary care. In addition, keywords in cluster 3 such as “mortality” and “hospitalization” remain hot topics focus on the ongoing interest in the efficacy of primary care of chronic diseases, keywords in cluster 4 such as “chronic diseases,” “comorbidity,” “multiple chronic conditions,” and “epidemiology” have consistently been key research areas in the epidemiological aspects of chronic diseases. “Mental health,” “chronic disease,” “health services research,” “qualitative research,” “service,” “chronic disease management,” “quality improvement,” “type 2 diabetes,” “physical activity,” “prevalence,” and “social determinants of health” have consistently been key research areas in PHC for chronic diseases, as shown in Figure 10C.

Furthermore, Figure 10D highlights that “mobile phone” and “primary healthcare” are the most popular keywords from 2021 to the present, and “social determinant” and “qualitative research” are the most popular keywords from 2022 to the present. The emergence of “mobile phone” as a recent keyword hotspot may relate to the increasing use of smartphones, which can enhance health monitoring efficiency and improve health surveillance and disease management models. The mention of “primary healthcare” indicates its critical role in advancing disease prevention efforts. “Primary healthcare” and “social determinant” as popular keywords underscore the significant impact of social factors on health, highlighting the need for a societal approach to strengthening primary healthcare. “Qualitative research” remains a key research type in the PHC for the chronic disease field. The inclusion of “food insecurity” as a keyword indicates that research in this field not only focuses on the diseases themselves but also addresses causative factors, suggesting that improving food security can effectively control disease occurrence.

The main theme of Figure 10A to D is the study of the current epidemiology of chronic diseases and the exploration of primary care management model effectiveness. Future research on innovative primary care management models will likely increase, with more scholars inclined to explore more efficient and precise health management methods through societal participation and internet empowerment. These efforts aim to reduce the incidence of suboptimal health and chronic diseases, improve the treatment and management of patients with multimorbidity, and enhance health-related quality of life.

## 5. Conclusions

This bibliometric analysis indicates that over the past 11 years, there has been slight fluctuation in the number of published articles in the PHC for the chronic disease research field. The overall trend shows an increase in publications, although there has been a decline in recent years. Nevertheless, the field of PHC for chronic diseases still holds promising research prospects. The United States and Canada are highly productive countries in this field, with the United States having a more significant influence. Clinical research on PHC for chronic diseases has garnered widespread attention. Interventional models and clinical management evidence for various chronic disease comorbidities in primary care have become potential hotspots. Notably, specific multimorbidity patterns may have common underlying pathogenic factors. It is essential to focus on researching these patterns to develop more targeted preventive measures, reduce their prevalence, and create new integrated approaches to manage these co-occurring conditions. This study helps scholars better understand the current state of PHC for chronic disease research from a macro perspective.

In summary, the significance of this study lies in several key aspects: first, the high prevalence of multimorbidity has become a major global public health challenge. The combination of chronic diseases, such as hypertension, diabetes, and arthritis, forms distinct patterns in different countries (e.g., the “metabolic-mental-joint” pattern), which may share common pathogenic mechanisms. Investigating these patterns can help uncover underlying biological or social factors, providing scientific evidence for the development of targeted prevention strategies and comprehensive management approaches. Second, PHC plays a central role in chronic disease management, with significant regional disparities in resource allocation and utilization efficiency. By analyzing the current state and intervention models of PHC (such as telemedicine and community engagement), the study can offer evidence to optimize resource distribution and improve the quality of care. In addition, emerging trends, such as the “social determinants of health” and the rise of “mobile health technologies” (e.g., disease monitoring via smartphones), highlight the need to integrate social factors and digital technologies to address the complexities of chronic disease management. Finally, bibliometric analysis reveals that current research is primarily focused on high-income countries (e.g., the United States and Canada), with limited data from low- and middle-income countries, underscoring the necessity of this research in promoting global health equity and fostering cross-regional collaboration. In conclusion, this study provides crucial insights into multimorbidity patterns, optimization of PHC management strategies, and the integration of emerging technologies, filling the knowledge gap in both mechanistic exploration and practical application of chronic disease interventions.

### 5.1. Limitations

The study included only English-language publications from the WoSCC and did not cover other databases (e.g., PubMed, Scopus) or research from non-English-speaking countries, potentially leading to the omission of regionally important findings, particularly those related to PHC practices in low- and middle-income countries. The absence of a standardized framework to address these differences may directly impact the comparability of the results. Furthermore, the reliance on quantitative indicators such as citation counts and publication numbers may underestimate the value of studies that are not frequently cited but still have practical significance. The analysis of emerging trends is limited by the time frame of the data collection, and the long-term impact of these trends will require further validation. In summary, while the study systematically identifies key issues and trends in chronic disease PHC, its conclusions are limited by the comprehensiveness of the data, methodological consistency, and predictions of long-term trends.

## Acknowledgments

First and foremost, I would like to express my sincere gratitude to all the scholars whose research works have laid the foundation for this bibliometric review. Their valuable contributions to the field of primary health care for chronic diseases have provided essential data and theoretical support for this study.

I am also deeply grateful to all members of the research team for their valuable collaboration, constructive discussions, and consistent support throughout the research and writing process. Their professional insights, rigorous attitudes, and selfless help have greatly assisted the team in overcoming difficulties and improving the quality of this manuscript.

In addition, I would like to thank the Web of Science Core Collection and SCI-E databases for providing the necessary literature data for this bibliometric analysis. Thanks also go to the developers of VOSviewer and CiteSpace software for their powerful tools that facilitated the data analysis process.

## Author contributions

**Data curation:** Ke Li, Chengquan Lu, Jian Wang.

**Methodology:** Ke Li, Jian Wang.

**Software:** Ke Li, Jian Wang.

**Validation:** Ke Li.

**Visualization:** Chengquan Lu, Jian Wang.

**Writing – Original Draft:** Ke Li.

**Writing – Review & Editing:** Chengquan Lu, Jian Wang.
